# Health Related Quality of Life in Patients with Type 2 Diabetes Mellitus in Iran: A National Survey

**DOI:** 10.1371/journal.pone.0044526

**Published:** 2012-08-30

**Authors:** Mehdi Javanbakht, Farid Abolhasani, Atefeh Mashayekhi, Hamid R. Baradaran, Younes Jahangiri noudeh

**Affiliations:** 1 Health Care Management and Economic Research Center, School of Health Care Management, Tehran University of Medical Sciences, Tehran, Iran; 2 School of Health Care Management and Information Sciences, Shiraz University of Medical Sciences, Shiraz, Iran; 3 Endocrinology and Mèolism Research Institute, Tehran University of Medical Sciences, Tehran, Iran; 4 Endocrine Research Center (Firouzgar), Institute of Endocrinology and Metabolism, Tehran University of Medical Sciences, Tehran, Iran; 5 Prevention of Metabolic Disorders Research Center, Research Institute for Endocrine Sciences Shahid Beheshti University of Medical Sciences, Tehran, Iran; Postgraduate Medical Institute & Hull York Medical School, University of Hull, United Kingdom

## Abstract

**Introduction:**

The aim of this study was to measure health-related quality of life (HRQoL) in Iranian people with Type 2 Diabetes Mellitus using two different measures and examines which socio-demographic and diabetes-related characteristics are associated with better quality of life based on a nationally distributed sample.

**Methods:**

A multi-stage cluster sampling method was used to select 3472 subjects as a part of Iranian surveillance of risk factors of non-communicable disease (ISRFNCD). EuroQol-5 Dimensions questionnaire (EQ-5D) and Visual Analog Scale (VAS) were employed to measure HRQoL. Binary logistic and Tobit regression models were used to investigate factors associated with EQ-5D results.

**Results:**

The mean age of subjects was 59.4 years (SD = 11.7), 61.3% were female and had 8.08 years (SD = 6.7) known duration of diabetes. The patients reported “some or extreme problems” most frequently in Pain/Discomfort (69.3%) and Anxiety/Depression (56.6%) dimensions of EQ-5D. The mean EQ-5D and VAS score were 0.70 (95% CI 0.69–0.71) and 56.8 (95% CI 56.15–57.5) respectively. Female gender, lower education, unemployment, long duration of diabetes, diabetes-related hospitalization in past years and having nephropathy and lower extremity lesions were associated with higher probabilities of reporting “some or extreme problems” in most dimensions of EQ-5D in binary logistic regression models. The same factors in addition to retinopathy were significantly associated with lower levels of HRQoL in Tobit regression analysis too.

**Conclusions:**

The study findings indicate that patients with diabetes in Iran suffer from relatively poor HRQoL. Therefore much more attention should be paid to main determinants of HRQoL to identify and implement appropriate policies for achieving better management of diabetes and ultimately improving the quality of life of diabetic patients in this region.

## Introduction

The risk of diabetes continues to increase worldwide due to population growth, aging, urbanization and increasing prevalence of physical inactivity and obesity [1]. The most recent data from International Diabetes Federation (IDF) indicate that the Middle East and North Africa (MENA) region has the highest rate of diabetes prevalence in the world. In this region about 12.5% of adults aged 20–79 years or 32.8 million people had diabetes in 2011 year and this number is expected to double in less than 20 years [2].

Type 2 diabetes mellitus (T2DM) is a complex and a serious chronic disease that impose a significant burden on patients and society in a term of morbidity and premature mortality[3], [4]. In the long term, diabetic patients have to face many complications. In addition to diabetes-related complications, episodes and fear of hypoglycemia and change in life style are the main cause of health-related quality of life (HRQoL) diminution [5].

Due to limited resource in health systems in worldwide demand for economic evaluations of health care programs, especially pharmaceuticals, is steadily increasing. One of the most important issues in this field is how to measure, value and incorporate changes in quality of life into the economic evaluation [6]. Moreover the importance of HRQoL in clinical research has been extensively discussed over recent years and there is an increasing recognition among clinicians and researchers that the impact of chronic illnesses and their treatments must be assessed in terms of their HRQoL in addition to more traditional measures of clinical outcomes – morbidity and mortality[7]–[9].

Heretofore a limited number of studies have been conducted in the Middle East to document the HRQoL of patients with T2DM [10]–[13]. Therefore the aim of this study was to measure HRQoL in Iranian patients with T2DM and examine which patient's socio-demographic and diabetes-related clinical characteristics are associated with better quality of life based on a nationally distributed sample.

## Methods

### Study Subjects and Sampling Design

This study conducted as a part of Iranian surveillance of risk factors of non-communicable disease system (ISRFNCD) that provided the demographic, anthropometric and biochemical characteristics on nationwide samples of Iranian adults. A multi-stage cluster sampling method was used to select 3918 patients with T2DM for study. Sampling frame was defined in 50 clusters for every 30 provinces. There is no distinction between rural and urban areas in samples, so that the samples were selected proportional to urban- rural population. All participants were visited by trained interviewers and were invited to participate by receiving informed consent. Subjects were eligible for the study if at the first they met WHO criteria (fasting plasma glucose ≥7.0 mmol/l (126 g/dl) or with a glucose tolerance test, two hours after the oral dose a plasma glucose ≥11.1 mmol/l (200 mg/dl)). Second were age 16 years or older and third were willing and able to give written informed consent and complete the questionnaire interview. Written informed consent also was obtained from guardian of individuals who were under 18 years old. Finally 3472 patients that completed two assigned questionnaires, included in the final analysis. The study has been approved by the ethics committee of the Iran's Ministry of Health (MOH)."

### Measures

Data were collected using two questionnaires, a socio-demographic and clinical history questionnaire and a validated Farsi version of HRQoL questionnaire. First one recorded details of age, gender, education level, Living condition, employment and marital status, disease duration, mode of treatment and related comorbidity. To determine the health status, the EQ-5D 3L questionnaire was used. It includes 5 questions, each assessing one of 5 dimensions of the HRQoL (Mobility (MO), Self-Care (SC), Usual Activities (UA), Pain/Discomfort (P/D) and Anxiety/Depression (A/D)). Each dimension has to be answered on a three–level scale (no problems, some or moderate problems, and extreme problems). The scales are given a score from 1 (no problem) to 3 (extreme problem) in each question; and finally the score digits are placed together to yield a 5-digit code for HRQoL of each patient. In this method, 243 (3 in power of 5) different codes are probable. EuroQol Group members have carried out researches mainly focused upon statistical modeling methods aimed at generating numerical values for each of 243 probable health states defined by EQ-5D. Value sets are commonly produced by valuing a selection of EQ-5D states and to extrapolate over the full set of states. In this study due to the absence of a locally appropriate set of values, as suggested by EuroQol Group the EQ-5D score was calculated using the UK VAS value set [14].The EQ-5D also contains a visual analog scale (VAS), measuring the subjects' perspectives of their quality of life level on a 100- point scale. The best state carries a score of 100 and the worst state a score of 0. This information can be used as a quantitative measure of health outcome as judged by the individual respondents. If patients had ability to answer the questions, they filled it personally; otherwise a trained interviewer collected the necessary data through face-to-face interviews with respondents.

### Statistical Analysis

The continuous variables were expressed as mean ± standard deviation and categorical variables as absolute numbers and percentages. Chi-square test was performed for the five dimensions of health status. As there were significant differences in EQ-5D and VAS scores according to age, sex, education level, employment status, diabetes duration and some comorbidity the mean values were adjusted by these parameters using analysis of covariance (ANCOVA). For all 5 dimensions level 2 and 3 on the EQ-5D dimensions were merged and thus dichotomized to “no problem” or “some or extreme problem”. We used logistic regression to obtain odds ratios (ORs) and 95% confidence intervals (95% CIs) for determinants of EQ-5D dimensions after adjustment for covariates. Confidence intervals were calculated using the bias-corrected accelerated (BCA) percentile bootstrapping method. Finally, a Tobit regression model was constructed to find factors that affected the HRQoL of the patients, using EQ-5D score as a dependent variable. The Tobit regression model is suitable for two reasons. First, the distribution of the dependent variable is skewed and censored at −0.053 and 1. Second, a considerable number of observations were at the upper limit of 1 (21.9%). All analyses were performed using STATA/SE 10.0 for Windows and SPSS Version 15.0.

## Results

The patients' characteristics have been described in details in table 1. The mean age of respondents was 59.4 years (SD = 11.7), 61.3% were female and the mean duration of diabetes was 8.08 years (SD = 6.7). About 80.8% was married, 82.3% had less than 6 grades education and 82.1% used combination therapy to control diabetes. Of the 3472 investigated patients, 32.6%, 21.6%, 40.4% and 10.7% reported that they had cardiovascular comorbidity, nephropathy, retinopathy and lower extremity lesions respectively.

**Table 1:** Characteristics of the patients.

Variables	Number (3472)	(%)
**Sex**		
Male	1344	38.7
Female	2128	61.3
**Age group**		
≤30 years	44	1.3
30–40 years	145	4.2
40–50 years	599	17.3
50–60 years	1042	30.0
60–70 years	1060	30.5
>70 years	582	16.8
**Marital status**		
Single	32	0.9
Married	2805	80.8
Divorced and loosed couple	635	18.3
**Education**		
≤6 grade	2857	82.3
6–12 grade	474	13.7
>12 grade	141	4.1
**Employment**		
Employed	690	19.9
Housewives+ students	1911	55
Unemployed	871	25.1
**Living area**		
Megacity (>1000000 population)	597	17.2
City (500000–1000000 population)	406	11.7
Small town (<500000)	2469	71.1
**Treatment**		
No treatment	427	12.3
Diet and exercise	195	5.6
Combination therapy (Medication + Diet and exercise)	2850	82.1
**Diabetes related hospitalization in past year**		
Yes	767	22.1
No	2705	77.9
**Diabetes duration**		
<5 years	1384	39.9
5–10 years	1428	41.1
>10 years	660	19
**Cardiovascular comorbidity**		
No	2246	67.4
Yes	1226	32.6
**Nephropathy comorbidity**		
No	2722	78.4
Yes	750	21.6
**Retinopathy comorbidity**		
No	2069	59.6
Yes	1403	40.4
**Lower extremity lesions**		
No	3100	89.3
Yes	372	10.7

### Dimensions of EQ-5D

In total 30%, 24.6%, 32.9%, 69.3% and 56.6% of the patients reported “some or extreme problems” in MO, SC, UA, P/D and A/D dimensions of EQ-5D respectively (Table 2). Examination of health status in both sex revealed that, the frequency of “some or extreme problems” were significantly higher in females for all dimensions (*P*<0.05). Patients in the 50 years had “some or extreme problems” and older group, those who divorced or lost their couple and those who had less than 6 grades education reported higher rate of “some or extreme problems” than patients in other groups in all dimensions. Same as subjects who were employed, the frequency of “some or extreme problems” for all dimensions were lower in patients who lived in region with populations 500000–1000000, compared to patients in other groups (*P*<0.05). As expected patients who had diabetes for longer time (5–10 and more than 10 years) reported significantly higher rate of had “some or extreme problems” in all dimensions except A/D. The frequency of “some or extreme problem” responses was significantly higher in subjects who had diabetes related hospitalization in past year (*P*<0.05). For the relationship between the presence of diabetic complications and health status, the frequency of “some or extreme problems” responses was significantly higher for all dimensions in patients with neuropathy and cardiovascular comorbidity and lower extremity lesions. Nevertheless, the presence of nephropathy and retinopathy comorbidity did not show a statistically significant relationship for the UA and MO and P/D dimensions respectively.

**Table 2:** Results of EQ-5D dimensions.

Variable	Mobility		Self-Care		Usual Activities		Pain/Discomfort		Anxiety/Depression	
	% of any problems	P-value	% of any problems	P-value	% of any problems	P-value	% of any problems	P-value	% of any problems	P-value
**Sex**										
Male	23.1	<0.001*	17.0	<0.001*	24.3	<0.001*	60.0	<0.001*	48.1	<0.001*
Female	34.3		29.4		38.3		75.2		62.0	
**Age group**										
≤30 years	6.8	<0.001*	6.8	<0.001*	4.5	<0.001*	36.4	0.001*	40.9	0.001*
30–40 years	8.3		4.8		11.0		50.3		57.2	
40–50 years	15.4		10.4		17.7		55.6		51.3	
50–60 years	23.1		17.2		25.6		68.1		55.1	
60–70 years	33.9		28.1		37.3		74.4		57.5	
>70 years	57.2		52.4		61.2		83.3		64.3	
**Marital status**										
Single	12.5	<0.001*	9.4	<0.001*	15.6	<0.001*	37.5	<0.001*	31.3	<0.001*
Married	26.5		21.0		29.2		66.7		54.8	
Divorced and loosed couple	46.3		41.4		50.1		82.5		66.1	
**Education**										
≤6 years	33.8	<0.001*	28.2	<0.001*	37.4	<0.001*	73.3	<0.001*	58.5	<0.001*
6–12 years	13.7		9.3		13.3		52.5		49.8	
>12 years	6.4		3.5		7.8		44.7		42.6	
**Employment**										
Employed	12.9	<0.001*	7.1	<0.001*	14.2	<0.001*	54.3	<0.001*	42.2	<0.001*
Housewives + Students	32.3		27.4		36.0		74.1		61.0	
Unemployed	38.3		32.4		40.9		70.5		58.6	
**Living area**										
Megacity (>1000000 population)	34.3	<0.001*	30.7	<0.001*	38.7	<0.001*	73.2	<0.001*	59.3	<0.001*
City (500000–1000000 population)	19.2		13.3		21.9		58.4		44.3	
Small town (< 500000)	30.7		25.0		33.3		70.1		58.0	
**Treatment**										
No treatment	21.2	<0.001*	18.2	<0.001*	21.2	<0.001*	57.6	<0.001*	47.0	0.001*
Diet and exercise	18.5		13.8		20.5		58.5		47.2	
Combination therapy (Medication + Diet and exercise)	32.2		26.3		35.2		72.1		58.7	
**Diabetes duration**										
<5 years	23.8	<0.001*	18.8	<0.001*	25.7	<0.001*	61.8	<0.001*	47.9	<0.001*
5–10 years	32.1		26.4		35.6		71.8		58.2	
>10 years	39.2		33.5		43.3		80.5		61.3	
**Diabetes related hospitalization in past year**										
Yes	46.4	<0.001*	41.5	<0.001*	50.7	<0.001*	82.4	<0.001*	69.9	<0.001*
No	25.3		19.8		27.8		65.6		52.9	
**Cardiovascular Comorbidity**										
No	27.6	<0.001*	22.0	<0.001*	29.9	<0.001*	68.4	<0.001*	54.9	<0.001*
Yes	38.5		32.3		42.3		76.4		63.6	
**Nephropathy Comorbidity**										
No	32.6	<0.001*	24.1	0.002*	34.5	0.219	67.2	<0.001*	55.5	<0.001*
Yes	25.6		30.0		31.9		84.7		65.8	
**Retinopathy Comorbidity**										
No	30.0	0.089	24.0	0.036*	31.9	0.003*	70.4	0.370	55.6	0.003*
Yes	32.8		27.3		37.0		71.9		60.9	
**Lower extremity lesions**										
No	29.3	<0.001*	23.9	<0.001*	32.5	<0.001*	69.7	<0.001*	57.0	0.012*
Yes	46.7		37.3		46.1		81.9		64.2	
Total	30.0		24.6		32.9		69.3		56.6	

Notes: *P*-value: chi-square test; No marks: not significant.

### EQ-5D and VAS Scores

The mean EQ-5D and VAS score were 0.70 (95% CI 0.69–0.71) and 56.8 (95% CI 56.15–57.5) respectively (table 3). The EQ-5D and VAS score were lower in females compared to male (0.67 vs. 0.74 for EQ-5D and 55.1 vs. 57.9 for VAS score). The patients who were older than 70 years, those who were divorced and loosed couple and where unemployed and those who had less than 6 grade education reported significantly lower EQ-5D and VAS scores compared to other groups. There wasn't Significant differences in EQ-5D score by treatment modality and living condition. We found that the patients who had diabetes related hospitalization in past year reported significantly lower EQ-5D and VAS scores (0.61 vs. 0.72 and 50.4 vs. 57.9). Those who had diabetes for longer time were reported lower EQ-5D and VAS scores. Finally those who had diabetes comorbidities reported significantly lower EQ-5D scores. The health status reported by EQ-5D scores was similar to that reported by VAS score, and there was a statistical relationship between EQ-5D score and VAS score (Spearman correlation test, *P* = 0.618; *P*<0.01).

**Table 3:** EQ-5D and Visual Analog Scale (VAS) scores.

Variable	EQ-5D Score				VAS			
	Mean	95% CI		P- value	Mean	95% CI		P- value
**Sex**								
Female	0.67	0.66	0.68	<0.001*	55.16	54.27	56.06	0.001*
Male	0.74	0.72	0.75		57.90	56.72	59.07	
**Age group**								
≤30 years	0.72	0.62	0.82	<0.001*	57.95	50.69	65.21	<0.001*
30–40 years	0.71	0.67	0.76		59.23	55.70	62.77	
40–50 years	0.73	0.71	0.75		59.21	57.46	60.96	
50–60 years	0.73	0.72	0.75		58.95	57.72	60.18	
60–70 years	0.69	0.68	0.71		55.35	54.14	56.57	
>70 years	0.60	0.58	0.62		49.31	47.58	51.04	
**Marital status**								
Single	0.69	0.59	0.80	<0.001*	52.14	44.13	60.15	0.001*
Married	0.71	0.70	0.72		56.90	56.14	57.66	
Divorced and loosed couple	0.65	0.63	0.67		53.39	51.69	55.09	
**Education**								
≤6 years	0.69	0.67	0.69	<0.001*	54.96	54.21	55.72	<0.001*
6–12 years	0.74	0.71	0.76		61.49	59.52	63.46	
>12 years	0.79	0.74	0.83		64.22	60.77	67.67	
**Employment**								
Employed	0.69	0.66	0.72	<0.001*	56.86	54.66	59.07	<0.001*
Housewives + students	0.74	0.72	0.76		58.08	56.67	59.49	
Unemployed	0.60	0.58	0.63		51.63	49.81	53.46	
**Living area**								
Megacity (>1000000 population)	0.678	.657	.700	0.146	54.56	52.93	56.20	0.01*
City (500000–1000000 population)	0.714	.678	.750		59.20	56.47	61.93	
Small town (<500000)	0.699	.688	.709		56.33	55.56	57.10	
**Treatment**								
No treatment	0.68	0.66	0.70	0.106	56.21	51.58	60.85	0.159
Diet and exercise	0.71	0.68	0.75		58.79	56.06	61.52	
Combination therapy (Medication + Diet and exercise)	0.70	0.69	0.71		56.02	55.31	56.72	
**Diabetes duration**								
<5 years	0.74	0.72	0.75	<0.001*	58.05	56.91	59.18	<0.001*
5–10 years	0.69	0.68	0.70		56.18	55.16	57.20	
>10 years	0.64	0.62	0.66		53.06	51.57	54.55	
**Diabetes related hospitalization in past** **year**								
Yes	0.61	0.59	0.63	<0.001*	50.48	49.08	51.88	<0.001*
No	0.72	0.71	0.73		57.96	57.19	58.73	
**Cardiovascular comorbidity**								
No	0.70	0.69	0.71	0.049*	56.39	55.55	57.22	0.449
Yes	0.68	0.67	0.70		55.80	54.58	57.03	
**Nephropathy comorbidity**								
No	0.66	0.64	0.68	<0.001*	56.85	56.09	57.62	<0.001*
Yes	0.71	0.70	0.72		53.86	52.41	55.31	
**Retinopathy comorbidity**								
No	0.69	0.68	0.70	0.042*	56.12	55.23	57.00	0.780
Yes	0.71	0.69	0.72		56.32	55.24	57.39	
**Lower extremity lesions**								
No	0.71	0.70	0.71	<0.001*	56.26	55.55	56.97	0.604
Yes	0.62	0.59	0.65		55.68	53.61	57.75	
Total	0.70	0.69	0.71		56.84	56.15	57.50	

Notes: EQ-5D and VAS scores: mean values adjusted by sex, age, education, marriage and employment status, diabetes duration and comorbidities; *P*-value: ANCOVA. No marks: not significant. VAS: visual analog scale.

### Regression models

Multivariate logistic regression models for each of the five dimensions of EQ-5D are shown in Table 4. Female reported more “some or extreme problems” with all dimensions of EQ-5D (MO; OR  = 2.24, SC; OR  = 2.61, UA; OR = 3.04, P/D; OR  = 2.29; A/D; OR  = 1.69). As indicated in table 4, the results showed that sex, education, employment status, diabetes related hospitalization in past year, duration of diabetes; nephropathy comorbidity and lower extremity lesions were significantly associated with reporting problems in the most dimensions of EQ-5D. Nonetheless marital status, living condition, treatment regime and retinopathy and cardiovascular comorbidity did not show significant results although the findings were in the expected direction.

**Table 4:** Results of multivariate logistic regression models.

Variable	Mobility		Self-Care		Usual Activities		Pain/Discomfort		Anxiety/Depression	
	OR	P-value	OR	P-value	OR	P-value	OR	P-value	OR	P-value
**Sex**										
Male	1.0 (ref.)		1.0 (ref.)		1.0 (ref.)		1.0 (ref.)		1.0 (ref.)	
Female	2.24	<0.001 *	2.61	<0.001 *	3.04	<0.001 *	2.29	<0.001 *	1.69	0.002*
**Age group**										
< = 30 years	1.0 (ref.)		1.0 (ref.)		1.0 (ref.)		1.0 (ref.)		1.0 (ref.)	
30–40 years	0.82	0.806	0.36	0.238	2.52	0.322	1.38	0.546	0.76	0.619
40–50 years	1.42	0.646	0.68	0.627	3.73	0.151	1.31	0.606	0.49	0.170
50–60 years	1.68	0.492	0.86	0.852	4.40	0.104	1.72	0.296	0.47	0.157
60–70 years	2.45	0.236	1.47	0.624	6.53	0.04*	2.14	0.144	0.45	0.133
>70 years	5.87	0.020*	3.66	0.102	15.48	0.003*	3.28	0.02	0.52	0.221
**Marital status**										
Single	1.0 (ref.)		1.0 (ref.)		1.0 (ref.)		1.0 (ref.)		1.0 (ref.)	
Married	0.65	0.541	0.87	0.860	0.32	0.105	1.46	0.493	3.65	0.024
Divorced and loosed couple	0.79	0.743	1.01	0.992	0.36	0.146	1.77	0.314	4.40	0.011*
**Education**										
<6 years	1.0 (ref.)		1.0 (ref.)		1.0 (ref.)		1.0 (ref.)		1.0 (ref.)	
6–12 years	0.49	<0.001*	0.47	<0.001 *	0.37	<0.001*	0.62	<0.001*	0.87	0.263
>12 years	0.20	<0.00*	0.15	<0.001	0.21	<0.001*	0.51	<0.001*	0.76	0.185
**Employment**										
Employed	1.0 (ref.)		1.0 (ref.)		1.0 (ref.)		1.0 (ref.)		1.0 (ref.)	
Housewives + students	1.22	0.372	1.52	0.092	0.93	0.749	0.92	0.696	1.22	0.283
Unemployed	2.44	<0.001*	3.11	<0.001*	2.26	<0.001*	1.38	0.014	1.66	<0.001*
**Treatment**										
No treatment	1.0 (ref.)		1.0 (ref.)		1.0 (ref.)		1.0 (ref.)		1.0 (ref.)	
Diet and exercise	0.71	0.361	0.61	0.230	0.84	0.652	1.04	0.897	0.97	0.908
Medication	1.01	0.972	0.86	0.678	1.15	0.667	1.35	.277	1.26	0.375
**Living area**										
Megacity (>1000000 population)	1.0 (ref.)		1.0 (ref.)		1.0 (ref.)		1.0 (ref.)		1.0 (ref.)	
City (500000–1000000 population)	0.94	0.771	0.61	0.049*	0.84	0.390	0.80	0.261	0.68	.032*
Small town (< 500000)	0.91	0.384	0.75	0.013*	0.84	0.104	0.80	0.060	0.84	.084
**Diabetes duration**										
<5 years	1.0 (ref.)		1.0 (ref.)		1.0 (ref.)		1.0 (ref.)		1.0 (ref.)	
5–10 years	1.27	0.02*	1.26	0.035*	1.33	0.004*	1.38	0.001*	1.38	<0.001*
>10 years	1.68	<0.001*	1.74	<0.001	1.81	<0.001*	2.10	<0.001*	1.50	<0.001*
**Diabetes related hospitalization in past year**										
Yes	1.0 (ref.)		1.0 (ref.)		1.0 (ref.)		1.0 (ref.)		1.0 (ref.)	
No	0.51	<0.001*	0.45	<0.001*	0.48	<0.001*	0.47	<0.001*	0.57	<0.001*
**Cardiovascular comorbidity**										
No	1.0 (ref.)		1.0 (ref.)		1.0 (ref.)		1.0 (ref.)		1.0 (ref.)	
Yes	1.16	0.121	1.19	0.102	1.22	0.042*	1.12	0.260	1.14	0.139
**Nephropathy comorbidity**										
No	1.0 (ref.)		1.0 (ref.)		1.0 (ref.)		1.0 (ref.)		1.0 (ref.)	
Yes	0.58	<0.001*	1.28	0.026*	0.76	0.009*	2.80	<0.001*	1.53	<0.001*
**Retinopathy comorbidity**										
No	1.0 (ref.)		1.0 (ref.)		1.0 (ref.)		1.0 (ref.)		1.0 (ref.)	
Yes	0.86	0.101	0.86	0.138	0.96	0.683	0.81	0.025*	1.05	0.545
**Lower extremity lesions**										
No	1.0 (ref.)		1.0 (ref.)		1.0 (ref.)		1.0 (ref.)		1.0 (ref.)	
Yes	2.02	<0.001*	1.73	<0.001*	1.54	.001*	1.64	0.002*	1.15	0.284

Notes: OR: Odds Ratio; Ref: reference group, No marks: not significant.

The results of Tobit regression model showed that the odds ratio of EQ-5D score compared to reference group was as follow; in female (0.85 95% CI 0.81–0.89), among who were older than 70 years (0.85 CI 0.73–1), in patients who had >12 grades education (1.17 CI 1.1–1.25), for unemployed subjects (0.89 CI 0.86–0.92), for those who had diabetes related hospitalization in past years (1.13 CI 1.1–1.16), among those who lived in Small town (1.03 CI 1–1.06), for those who had diabetes more than 10 years (0.89 CI 0.86–0.92), in patients with nephropathy comorbidity (0.93 CI 0.91–0.96) and for those with lower extremity lesions were (0. 9 CI 0.87–0.94) (table 5).

**Table 5:** Results of Tobit regression model.

Indicator	EQ-5D Score			
	Odds Ratio	95% CI		P- value
**Sex**				
Male	1 (Ref.)			
Female	0.85	0.81	0.89	<0.001*
**Age group**				
< = 30 years	1 (Ref.)			
30–40 years	0.96	0.82	1.13	0.632
40–50 years	1.00	0.86	1.16	0.961
50–60 years	0.99	0.85	1.15	0.884
60–70 years	0.95	0.82	1.10	0.500
>70 years	0.85	0.73	1.00	0.044*
**Marital Status**				
Single	1 (Ref.)			
Married	0.92	0.78	1.08	0.295
Divorced and loosed couple	0.88	0.74	1.03	0.117
**Education**				
<6 years	1 (Ref.)			
6–12 years	1.09	1.05	1.13	<0.001*
>12 years	1.17	1.10	1.25	<0.001*
**Employment**				
Employed	1 (Ref.)			
Housewives+ students	1.03	0.98	1.09	0.266
Unemployed	0.89	0.86	0.92	<0.001*
**Treatment**				
No treatment	1 (Ref.)			
Diet and exercise	1.07	0.98	1.16	0.161
Medication	1.02	0.94	1.10	0.613
**Living area**				
Megacity (>1000000 population)	1 (Ref.)			
City (500000–1000000 population)	1.06	1.00	1.11	0.043*
Small town (<500000)	1.03	1.00	1.06	0.039*
**Diabetes duration**				
<5 years	1 (Ref.)			
5–10 years	0.94	0.92	0.97	<0.001*
>10 years	0.89	0.86	0.92	<0.001*
**Diabetes related hospitalization in past year**				
Yes	1 (Ref.)			
No	1.13	1.10	1.16	<0.001*
**Cardiovascular comorbidity**				
No	1 (Ref.)			
Yes	0.98	0.95	1.00	0.052
**Nephropathy comorbidity**				
No	1 (Ref.)			
Yes	0.93	0.91	0.96	<0.001*
**Retinopathy comorbidity**				
No	1 (Ref.)			
Yes	1.03	1.00	1.05	0.029*
**Lower extremity lesions**				
No	1 (Ref.)			
Yes	0.90	0.87	0.94	<0.001*

Notes: No marks: not significant.

## Discussion

To our best knowledge it seems that this is the first population-based study to investigate HRQoL of patients with T2DM in MENA region; where that has highest rate of diabetes prevalence in world. We measured the health status by EQ-5D and calculated the EQ-5D score, to investigate the relationship between EQ-5D and VAS scores and patient characteristics that are associated with HRQoL. Synthesis of literature indicated that the EQ-5D has been used to measure HRQoL of diabetic patients in different countries [15]–[19]. The EQ-5D has been used in the UK Prospective Diabetes Study (UKPDS) to determine the effects of therapy, complications, and hypoglycemic episodes on HRQoL in patients with T2DM too [20]. We used EQ-5D for two reasons, first using the EQ-5D instrument able us to transform the utility scores into quality-adjusted life years for use in economic evaluations of new therapies. Second the shorter completion time compared with other generic instruments.

We showed that the patients reported “some or extreme problems” in a range of 24.6%–69.3% with highest rate for P/D and A/D dimensions. Review of literature indicated that problems in the P/D and A/D dimensions were most frequently reported [17], [21]. Although Sakamaki and his colleague showed that diabetic patients had reported more problem with Mo and P/D dimensions[19].

We concluded that mean EQ-5D score in Iranian patients with T2DM was 0.70 while investigation of studies that used same instruments in Japanese, Canadian, Korean and Norwegian patients were 0.862, 0.75, 0.9 and 0.85 respectively[15], [17], [19], [22]. Many socioeconomic and healthcare system related factors influence HRQoL of diabetic patients; therefore comparison of results should be interpreted with caution. Some of these differences could be explained by difference in main characteristics of studied subjects such as; mean age, duration of diabetes and comorbidities. Moreover since diabetes produces few symptoms and is initially not life threatening, many people, particularly in developing countries, often do not seek medical attention until other incapacitating symptoms or complications develop [23], [24]. Delay in diagnosis can directly increase complications and then lead to higher diminution of patient's HRQoL.

We have concluded that mean EQ-5D score was lower in female compared to male. This finding is in accordance with other studies of similar patients [15], [18], [19], [25], [26]. Better social life and physical activity of men in developing countries like Iran might contribute to higher level of satisfaction; also women tend to be more expressive and thus are more likely to complain about a poor quality of life. Moreover studies have shown that men were more confident of their ability to control diabetes and reported a higher quality of life and were less likely to get depression or anxiety compared to women [27]. Our results showed that increased age was associated with lower HRQoL. Other studies have reported same finding [15], [26], [28], [29]. Surprisingly, in contrast to these studies and our findings, Daria and his colleague showed that increased age was associated with better HRQoL [22]. Our results showed that being female, less educated and unemployed, having diabetes related hospitalization in past years, living in bigger cities, having diabetes for longer time, having nephropathy and retinopathy comorbidity and lower extremity lesions were significantly associated with lower EQ-5D scores. These results are in accordance with the findings reported by Rubin and his colleague, who systematically analyzed all recent literature on diabetes and quality of life [30]. Moreover Redekop et al found that older age, female sex, insulin therapy, presence of complications, and obesity were associated with a lower HRQoL [18].

Education level had a linear relationship with quality of life. As the educational level increased the quality of life increased. This could be due to they will have a better understanding of the disease, its effect on them, and will avail themselves the best treatment they can afford. Duration of disease has shown a variable that affect HRQoL negatively. In two Finnish studies duration was associated with reduced HRQoL, particularly physical functioning [31], [32]. Similarly Arghese et al and Saito his colleague in two different studies showed that as duration of the disease increases, the health of the patient will gradually worsen depending on his control of diabetes [33], [34]. Conversely number of studies conducted in different countries found no association between disease duration and HRQoL that was measured by generic and specific instruments [16], [18], [35], [36].

Our results showed that there was no detectable difference in EQ-5D score among patients in different treatment group. Speight and his colleague concluded that the EQ-5D may capture differences due to diabetes related complications it will not necessarily be able to capture differences across treatment regimens. This is because the extent to which a given treatment is considered flexible or convenient will not affect quality of health but may affect aspects of quality of life, such as social or working life [37]. Similarly other study in Japan found same results and suggest that EQ-5D is less sensitive than disease-specific scales to treatment modality and should be used in combination with the disease-specific scale for clinical evaluations [19].

We used ordinal logistic regression analysis to create five models, one for each dimension, to determine which patient characteristics were associated with reporting problems in these dimensions. Although the composition varied somewhat between models, several patient characteristics appeared in at least three of the five models. Solli and his colleague concluded that age, impaired vision, stroke, neuropathy, body mass index, sex, limitations at work, number of hospital admissions during the previous 6 months, fear of hypoglycemia and receiving help from others were statistically significant determinants of reporting problem in different dimensions of EQ-5D [17].

The results of Tobit regression model showed that female sex, less educated and unemployed status, having diabetes related hospitalization in past years, living in bigger cities, having diabetes for longer time, having nephropathy and retinopathy comorbidity and lower extremity lesions were significantly associated with lower EQ-5D scores. Solli and his colleague showed that stroke, neuropathy, disability pension, receiving help from others, fear of hypoglycaemia and limitations at work were significantly associated with EQ-5D score, where a linear regression model was used [17].

There are several limitations of our study that merit consideration in interpreting results. First, although diabetes complications were closely related to individual HRQoL level [15], [17], [18], we did not assess all diabetic complications effect on HRQoL because ISRFNCD is not a survey only for diabetes and does not provide all data related to diabetes complications. We also lacked information on potentially useful clinical variables. Furthermore our study was performed at one point in time, and fluctuations are likely to occur if HRQoL measured at multiple points in time. Since this is a cross-sectional study, the observed associations are not necessarily causal.

The study findings indicate that patients with diabetes in Iran suffer from relatively poor HRQoL. Therefore much more attention should be paid to main determinants of HRQoL to identify and implement appropriate policies for achieving better management of diabetes and ultimately improving the quality of life of diabetic patients in this region. Because of the relationships found in this study as well as in other studies, it is reasonable to conclude that any efforts to avoid or postpone obesity, inactivity, smoking, and development of complications will enhance HRQoL and thereby improve healthy life expectancy.
